# The grade of instability in fragility fractures of the pelvis correlates with impaired early mobilization

**DOI:** 10.1007/s00068-022-01933-y

**Published:** 2022-03-13

**Authors:** Leon Marcel Faust, Alexander Martin Keppler, Eduardo Suero, Johannes Gleich, Leonard Lisitano, Wolfgang Böcker, Carl Neuerburg, Daniel Pfeufer

**Affiliations:** 1 Department of Orthopaedics and Trauma Surgery, Musculoskeletal University Center Munich (MUM), University Hospital, LMU Munich, Munich, Germany; 2 Department of Trauma, Orthopaedic, Plastic and Hand Surgery, University Hospital of Augsburg, Augsburg, Germany

**Keywords:** Orthogeriatrics, Pelvic fracture, Fragility fractures of the pelvis, Wearables, Gait analysis, Mobility

## Abstract

**Purpose:**

This study aimed to investigate whether gait patterns of patients with fragility fractures of the pelvis (FFP) comply with the grade of fracture instability, defined by radiological patterns.

**Patients and methods:**

This prospective, single-center, observational study included 39 patients with an FFP. Gait analysis was performed with a wearable insole force sensor (Loadsol® by Novel, Munich, Germany) 4–7 days after admission. Patients were divided in two groups: Group A included FFP type 1 fractures, which affect the anterior pelvic ring only, Group B contained FFP type 2–4 fractures with an involvement of the posterior pelvic ring. Primary outcome parameter was the FTI ratio (force–time integral (N*s)).

**Results:**

The mean age was 85.08 years (SD ± 6.45), 94.9% (37/39) of the patients were female. The most common fracture type was an FFP 2b (64.1%, 25/39). Group A showed a significantly higher FTI ratio (45.12%, SD ± 4.19%) than Group B (38.45%, SD ± 5.97%, *p* = 0.002). Further, a significant correlation of the FTI ratio and the average (*r* = 0.570, *p* < 0.001) and maximum (*r* = 0.394, *p* = 0.013) peak force was observed.

**Conclusion:**

The gait pattern of patients with an FFP type 2–4 was more imbalanced than of patients with an FFP type 1 fracture. These findings match with the radiological classification of FFP, which indicates higher instability, when the posterior pelvis is affected. Gait analysis might offer earlier functional diagnostics and may accelerate the treatment decision with shorter periods of immobility in future. Especially in cross-border cases, early gait analysis could be beneficial to clarify the indication for or against surgery.

## Introduction

Facing an aging global population, treating orthogeriatric patients has become a major burden in orthopedic surgery. Fragility fractures of the pelvis (FFP) are a rapidly emerging entity in these patients, and several observational studies have revealed an increased incidence of FFP over the last decade [1–3].

This fracture type largely differs from pelvic fractures in younger patients, which are mostly caused by high-energy trauma. FFP are caused by low-energy trauma or occur as spontaneous insufficiency fractures, and can be classified as osteoporotic fractures [4–6]. Rommens et al. developed the FFP classification system, categorizing the fractures by radiomorphological and clinical criteria [4]. FFP 1 fractures are characterized by a unilateral or bilateral anterior pelvic fracture. Following Rommens’ clinical guidance, FFP 1 fractures do not require surgery, but still pose a threat to the patients’ health condition [7]. FFP 2 fractures are moderately unstable and defined by a unilateral, non-displaced fracture of the posterior ring, with or without an anterior pelvic lesion. The choice of treatment is complex. In case of prolonged immobility and insufficient pain relief, operative treatment should be considered [8]. FFP 3 fractures encompass unilateral, displaced fractures of the posterior pelvic ring, with simultaneous affection of the anterior pelvic ring and a high degree of instability. FFP 4 fractures are bilateral, displaced lesions of the posterior pelvic ring with the highest grade of instability. Anterior pelvic affection may occur in FFP 4. For both FFP 3 and 4, surgical treatment is advised [8]. Recommendations for fixation techniques are currently based on biomechanical and retrospective studies. There are several options such as iliosacral screw osteosynthesis, trans-sacral bar osteosynthesis, lumbopelvic fixation, or sacroplasty [8–10]. Recent studies showed that percutaneous treatment is associated with fewer complications, shorter length of hospital stay and lower mortality than open surgical techniques [11, 12]. Accurate diagnostics in FFP are challenging: in patients with anterior pelvic lesions, involvement of the posterior pelvis is frequently missed [13]. Usually, patients admitted to the emergency department first undergo a pelvic X-ray followed by a CT scan, although pelvic MRI scans are considered as the gold standard in radiologic diagnostics of the pelvis [14]. It was also shown that MRI is the most sensitive method to find non-displaced pelvic fractures in patients with low bone density [15]. Still, MRI scans are often only performed in case of prolonged pain and immobility, due to high costs and limited availability. Recent studies pointed out that dual-energy CT scans could be a promising method with up to 100% sensitivity and specificity in fracture diagnostics [16]. Besides radiologic diagnostics, it is necessary to evaluate the functional mobility of FFP patients. Immobilizing fractures in orthogeriatric patients bear a high risk of complications, such as urinary tract infections, delirium, pneumonia, decubital ulcers or prolonged hospitalization [17, 18]. Additionally, even short periods of muscle disuse can result in vast muscular atrophy [19]. Therefore, a delayed therapeutic decision with prolonged immobility may result in severe consequences [20]. Precise gait analysis with wearable sensors could help to identify patients with severely impaired mobility. Gait analysis is a novel functional diagnostic method to assess weight-bearing of the lower extremity. Wearable insole force sensors have proven to be a feasible tool for gait analysis in orthogeriatric patients [21, 22]. Moreover, they have shown to be capable of measuring differences in weight-bearing among FFP patients [23].

The FFP classification complies with the radiological instability of fracture patterns [4]. We hypothesized, that the impairment in gait patterns in FFP patients is in line with the radiological degree of instability and may bring forth objective gait parameters that can be integrated into the treatment decision.

## Patients and methods

### Study design and participants

**Fig. 1:**
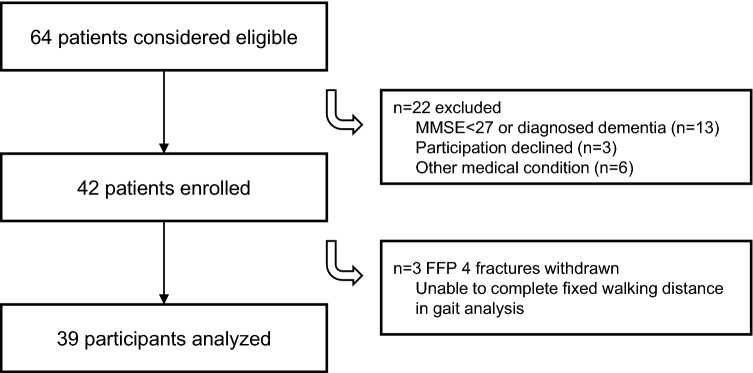
Patient selection process with reasons for primary exclusion and withdrawal in the further course of the study

Patients aged > 65 years, treated with an FFP 1–4 in a certified orthogeriatric unit were considered eligible for enrollment in this prospective observational trial (level of evidence 2). The participants were recruited between October 2019 and April 2021. An ad hoc power analysis was performed with powerandsamplesize.com (HyLown Consulting LLC, Atlanta, USA) based on data from an internal pilot study. It showed that patients with an FFP 1 had a mean FTI ratio of 44.99% (SD ± 5.44) and patients with an FFP 2–4 had a mean FTI ratio of 39.23% (SD ± 6.01). To achieve 80% power at a level of significance *p* < 0.05, a required number of 19 patients per group was calculated. Criteria for exclusion were: a Minimal Mental State Exam (MMSE) score lower than 27, language barriers, patients suffering from an additional fracture (even of the upper extremity), and patients with a pre-existing neurological or musculoskeletal condition that affects gait and balance. Two senior consultants classified the fractures according to the FFP classification by Rommens et al. [4]. At first, they classified the fractures independently and in case of discordance both consultants reviewed the imaging together to find an agreement. Primary conservative treatment was considered failed when immobility persisted for 4–7 days after admission. The selection process is outlined in Fig. 1.

### Gait analysis procedure

**Fig. 2:**
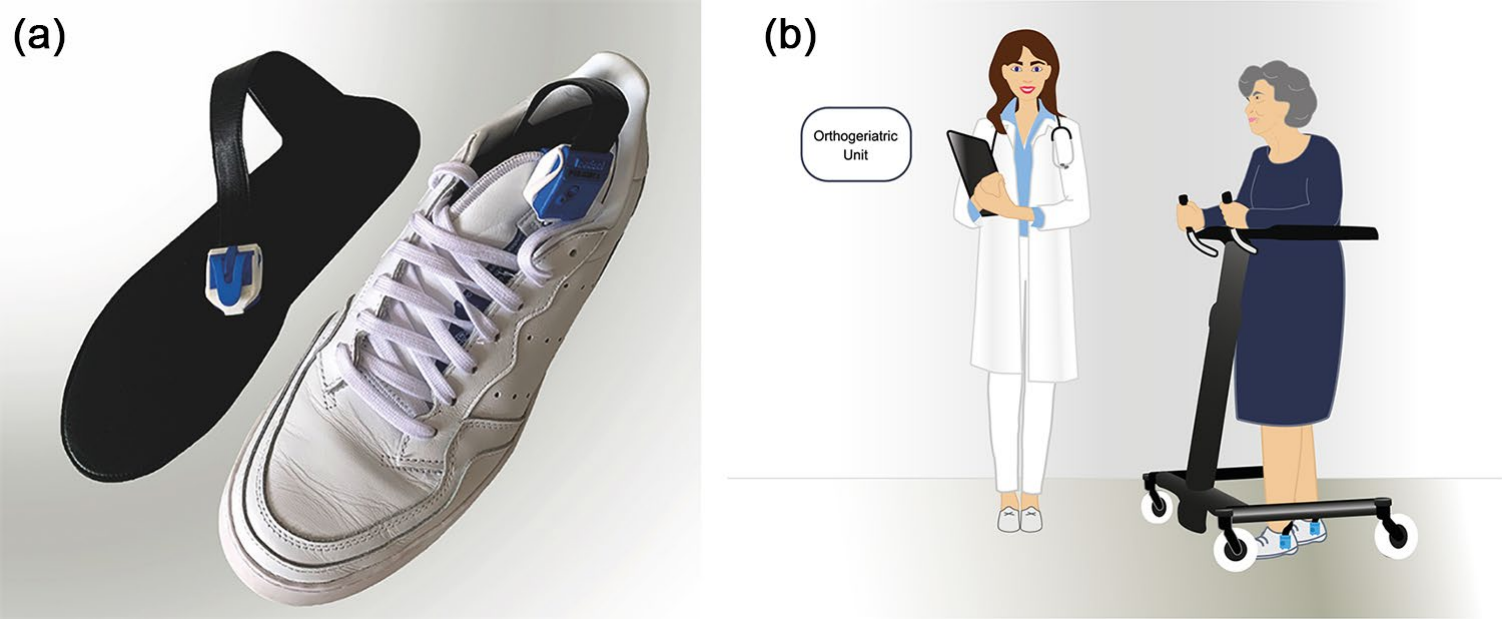
**a** The Loadsol® insole is fitted into the patients’ shoes. **b** Illustration of the measurement process. The patient is mobilized with a walker and a member of the research team is collecting the gait data using a tablet

The wearable insole force sensor Loadsol® by Novel (Munich, Germany) was used for gait analysis. Previous studies have shown that the Loadsol® is an accurate and valid device for ground reaction force measurement [24, 25]. 4–7 days after admission, all patients were mobilized using a height-adjustable walker with armrests. A pair of Loadsol® insoles matching the patients’ shoe size was placed in the patients’ shoes (see Fig. 2a, b). The insoles measure the force between the full plantar surface of the foot and the shoe. The sensors were connected to an iPad (Apple Inc., Cupertino, CA, USA) via Bluetooth and transmitted the records in real time. The participants were instructed to walk a fixed distance of 10 m back and forth. All patients received standardized pain medication in compliance with the WHO analgesic ladder and none of the patients were treated with a local pain catheter on the day of measurement.

Gait analysis was performed regardless of the choice of treatment. The decision for surgical treatment was made in a multidisciplinary team with proficient consultants, a geriatrician, physiotherapists and in close consultation with the patient. Therefore, data collected from surgically treated patients was collected preoperatively.

### Parameters

**Fig. 3:**
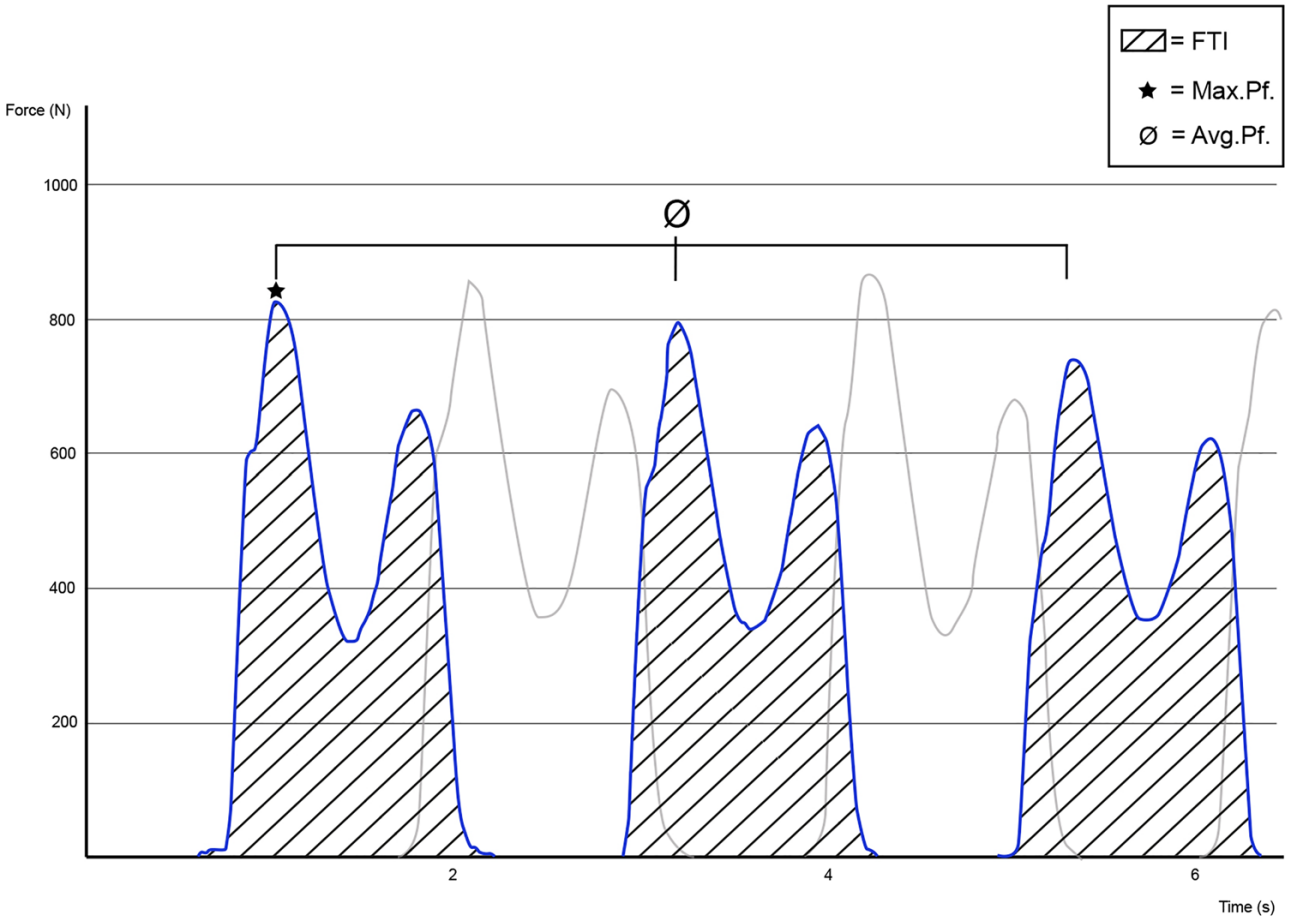
Visualization of the force–time curve recorded during gait analysis. Three exemplary steps are depicted for both feet, (limb 1: blue, limb 2: gray). Y-axis: force in newton, X-axis: time in seconds. The legend shows the three main parameters: force–time integral (FTI), maximum peak force (Max. Pf.) and average peak force (Avg. Pf.) and how they are marked in the graph

There were three parameters used for statistical analysis (Fig. 3). The primary outcome parameter was the FTI ratio. FTI is defined as force–time-integral (N*s) and represents the area under the curve, measured for each foot separately. The FTI ratio is calculated as the FTI of the affected limb divided by the summed-up FTI values of both limbs. The result is expressed as a percentage, with an optimum value of 50%, meaning that both feet were loaded perfectly balanced.

The average peak force (Avg. Pf.) in newton is the average of all maximum force values recorded for each foot separately. The Avg. Pf. was divided by the patients’ total bodyweight in Newton, to receive the percent Avg. Pf. The single highest force applied to each foot is the maximum peak force (Max. Pf.). It was also divided by the body weight (N), to receive the percent Max. Pf. The 11-point numeric rating scale was used to assess pain. Parker Mobility Score (PMS), Barthel Index (BI) and Charlson Comorbidity Index (CCI) were assessed for each patient.

### Statistical analysis

Descriptive data on patient characteristics were generated for the whole cohort and the different FFP types individually. Two groups were defined based on the assumption that a lesion of the posterior pelvic ring leads to a greater impairment of gait parameters than a fracture of the anterior pelvic ring only. Group A encompasses patients with an FFP type 1, while FFP type 2, 3 and 4 were assigned to Group B. To check for normal distribution, the Shapiro–Wilk test was performed, followed by a Mann–Whitney *U* test to compare the means of both groups. Bivariate Pearson correlation was used to measure the strength and direction of the relationship of the gait parameters mentioned. There was no missing data in the displayed statistical analysis. The level of significance was set at *p* < 0.05. Statistical analysis was performed with IBM SPSS Statistics Version 25 (IBM Germany GmbH, Ehingen, Germany). Illustrations were created using Adobe Illustrator® Version CC 2021 (Adobe Systems Software Ireland Limited, Dublin, Ireland).

## Results

### Population characteristics

In total, 39 patients met the inclusion criteria and completed gait analysis according to the study protocol. 94.9% of the participants were female (37/39), the mean age was 85.08 years (SD ± 6.45, range 69–98 years). Total body weight averaged 60.04 kg (SD ± 9.04), with a mean BMI of 22.28 kg/m^2^ (SD ± 3.37). The median CCI value was 5 (IQR 4–6) and the median MMSE score was 29 (IQR 28–30). The analysis involved *n* = 6 FFP 1a (15.4%), *n* = 2 FFP 1b (5.1), *n* = 25 FFP 2b (64.1%), *n* = 1 FFP 2c (2.6%), *n* = 1 FFP 3c (2.6%), *n* = 1 FFP 4b (2.6%) and *n* = 3 FFP 4c (7.7%).

### Comparison of FFP subtypes

First, we compared the descriptive statistics generated for all four FFP subtypes individually. A comparison of PMS, BI, CCI and gait parameters among the four FFP subtypes can be seen in Table 1. It shows that the load put on the affected limb differs between the FFP subtypes, as represented by the percent Avg. Pf., the percent Max. Pf. and the FTI ratio. Minor differences can be seen between the FFP subtypes in view of BI, PMS and CCI.

**Table 1:** Descriptive statistics displayed for each FFP subtype

	FFP 1	FFP 2	FFP 3	FFP 4
BI^a^ before injury	100 (IQR 90–100)	92.5 (IQR 80–100)	100	97.5 (IQR 83.75–100)
BI^a^ at inpatient stay	65 (IQR 40–75)	47.5 (IQR 33.75–61.25)	75	55 (IQR 22.5–80)
PMS^a^ before injury	9 (IQR 9–9)	6.5 (IQR 5–9)	9	7 (IQR 6.25–7.75)
CCI	4 (IQR 4–5.75)	5 (IQR 4–6)	5	5 (IQR 4–9)
Avg. Pf. (% of body weight)	71.78 ± 23.36	53.83 ± 16.12	54.09	54.01 ± 21.82
Max. Pf. (% of body weight)	84.79 ± 21.20	68.93 ± 15.02	63.15	73.82 ± 14.34
FTI ratio (%)	45.12 ± 4.19	38.25 ± 6.03	41.67	38.97 ± 7.05

Abbreviations: *BI* Barthel Index, *PMS* Parker Mobility Score, *CCI* Charlson Comorbidity Index, *Avg. Pf.* average peak force, *Max. Pf.* maximum peak force, *FTI* force–time integral

^a^BI and PMS were calculated for the time at home before the injury and hospitalization. BI was obtained for the inpatient stay as well

Table 2 shows a comparison of the descriptive statistics between patients that received conservative treatment (*n* = 25; with *n* = 8 FFP 1, *n* = 16 FFP 2, *n* = 1 FFP3) and patients that underwent surgery (*n* = 14; with *n* = 10 FFP 2 and *n* = 4 FFP 4) during their inpatient stay.

**Table 2:** Descriptive statistics displayed depending on the treatment

	Conservative Treatment (*n* = 25)	Surgical Treatment (*n* = 14)
BI^a^ before injury	100 (IQR 85–100)	92.5 (IQR 78.25–100)
BI^a^ at inpatient stay	55 (IQR 42.5–70)	45 (IQR 25–65)
PMS^a^ before injury	9 (IQR 5.5–9)	6.5 (IQR 5.75–9)
CCI	5 (IQR 4–6)	4.5 (IQR 4–6)
Avg. Pf. (% of bodyweight)	63.59 ± 16.52	46.72 ± 18.96
Max. Pf. (% of body weight)	75.87 ± 15.87	66.57 ± 17.99
FTI ratio (%)	40.61 ± 5.95	38.42 ± 6.70

Abbreviations: *BI* Barthel Index, *PMS* Parker Mobility Score, *CCI* Charlson Comorbidity Index, *Avg. Pf.* average peak force, *Max. Pf.* maximum peak force, *FTI* force–time integral

^a^BI and PMS were calculated for the time at home before the injury and hospitalization. BI was obtained for the inpatient stay as well

### Main finding

**Fig. 4:**
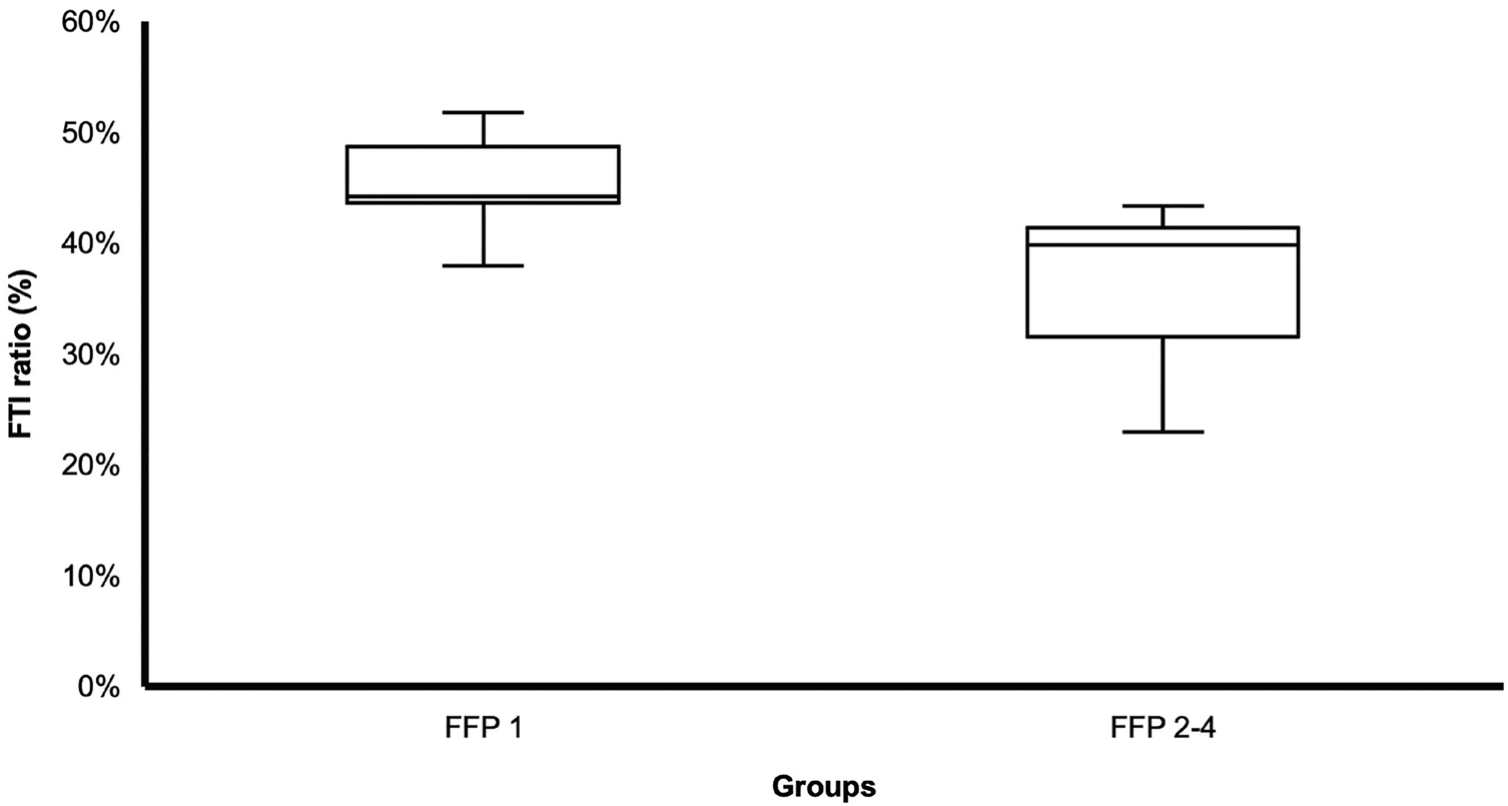
Box plot of Group A (FFP 1) and Group B (FFP 2–4), *p* = 0.002. The FTI ratio (%) is shown on the Y-axis

Patients with an FFP 1 (Group A) showed a significantly higher FTI ratio than patients with an FFP 2–4 (Group B). The mean FTI ratio of Group A was 45.12% (SD ± 4.19%), while Group B averaged 38.45% (SD ± 5.97%; *p* = 0.002) (Fig. 4).

The median PMS score in Group A was 4.00 (IQR 1.00) and 2.00 (IQR 3.00) in Group B (*U* = 55.00, *p* = 0.044). Regarding the BI, a median of 65.00 (IQR 35.00) in Group A and a median of 50.00 (IQR 30.00) in Group B (*U* = 70.00, *p* = 0.156) were observed.

Bivariate correlation analysis presented significant correlation of the percent Avg. Pf., the percent Max. Pf. and the FTI ratio. The FTI ratio showed a strong positive correlation with the percent Avg. Pf. and a moderate positive correlation with the percent Max. Pf. (see Table 3).

**Table 3:** Correlation matrix of gait parameters

		% Avg. Pf	% Max. Pf
FTI ratio	Coefficient (r)	0.570 (strong)	0.394 (moderate)
	*p* value	< *0.001*	*0.013*

Statistically significant *p* values are in italics (*p* < 0.05)

## Discussion

This study aimed to investigate whether radiologically defined patterns of instability in FFP could be reproduced with a wearable insole force sensor-based gait analysis. The most relevant finding is that patients with a lesion of the posterior pelvic ring average a lower FTI ratio than patients with a lesion of the anterior pelvic ring only, indicating a more imbalanced gait pattern in patients with an FFP 2–4.

Previous investigations of gait patterns in FFP already showed that the Loadsol® is capable of measuring differences in weight-bearing when comparing the FFP subtypes [23]. While previous research analyzed gait using the Avg. and the Max. Pf., this current study focused on the FTI ratio as a new parameter. The FTI (area under the curve, N*s) includes time and represents the complete gait cycle of every step taken. The Avg. and Max. Pf. on the other hand both express peak ground reaction forces (N) applied to the respective foot during the stance phase. Therefore, the FTI ratio broadens the methodological approach to the examination of gait patterns in FFP patients. The FTI ratio indicates gait asymmetry, which can be interpreted as a measure of gait quality. Higher gait asymmetry is positively correlated to fall risk and dependency in activities of daily living [26].

The FTI ratio and the Avg. Pf. demonstrated a strong correlation (*r* = 0.57, *p* ≤ 0.001), while the FTI ratio and the Max. Pf. showed moderate correlation (*r* = 0.394, *p* = 0.013). Good agreement of all three gait parameters is a necessary foundation to use them for further gait analysis of FFP patients.

Biomechanical and anatomical characteristics of the pelvic ring are key factors for the identified differences between fractures limited to the anterior pelvis (Group A) and those with a lesion of the posterior pelvis (Group B). Finite element (FE) analyses in a model based on walking have shown that a higher portion of the load is transferred through the posterior pelvis in comparison to the anterior part [27]. Fractures in the posterior part of the pelvic ring cause a higher degree of damage to the structural integrity of the pelvic ring [28]. This substantiates our finding that a lesion of the posterior pelvis causes a more asymmetric gait pattern and thus a higher impairment of mobility. Yet, unilateral anterior pelvic fractures also affect the pelvic load distribution, as another FE analysis study showed [29]. In case of a fracture of both pubic rami, it was found that the load is transferred through the posterior pelvis entirely, which causes increased stress on the posterior structures.

Gait analysis is a novel and progressive approach to examine FFP. The results of this study show that there were no significant differences of the BI between the defined subgroups, while the PMS showed a weak significant difference. In contrast, significant changes in the actual weight-bearing of the affected limb could be observed via gait analysis. That emphasizes the added value of gait analysis in research of FFP, considering that previous studies primarily used ADL and standardized mobility scores to investigate patient mobility [7, 30].

In this study, patients with an FFP 2 made up the largest share of all subtypes. FFP 3 represent the smallest group of all subtypes. This pattern is in line with earlier studies of other research groups [4, 8, 31]. We are aware that there are a few limitations to our study. The small population size is a major limitation. Further, the number of patients included in the two groups compared show an unequal distribution, as our analysis focused on the comparison of fractures of the anterior pelvis, including only one subtype, and those with a fracture of the posterior pelvis, including three subtypes. Therefore, the results should be interpreted with caution. The criteria for exclusion were defined to avoid selection bias, e.g., due to patients with cognitive impairment or preexisting conditions altering gait. The non-randomized trial design may cause selection bias. Given the elderly orthogeriatric population, it was inevitable that several patients were disqualified from participation. Moreover, multimorbidity is highly prevalent in this population, which might entail a contraindication for surgery, regardless of the fracture type or mobility. Unfortunately, the population size does not allow to further differentiate between all FFP subtypes. Second, we only examined patients during early mobilization in the first 4–7 days after admission. Nevertheless, this is a crucial period, as early mobilization is an essential factor affecting the treatment decision [4, 8]. However, follow-up investigations of gait during the inpatient stay and ambulant aftercare should be promoted. In addition, three enrolled patients could not finish the defined distance required for gait analysis. All three had an FFP 4, so it might be assumed they would fit into the present findings, considering their vastly impaired mobility. There are various wearable motion sensors as an alternative to the Loadsol®, with different sensor technology and therefore may provide additional insights with different gait parameters [32, 33].

## Conclusion

The present analysis represents a preliminary approach to introduce functional diagnostics with insole force sensors to assess the impairment of mobility in elderly patients with FFP. Gait analysis assessed with the FTI ratio has shown a significant correlation with the radiographic fracture pattern of FFP. In the future, wearable sensors might help to reduce the duration of immobilization and improve the choice of treatment in FFP. The consideration is particularly difficult for FFP 2 patients [8]. Using gait analysis as an additional diagnostic tool might enhance decision-making in these patients.

The results have to be interpreted carefully, considering the small number of participants and the non-randomized study design. Our hypothesis is thereby confirmed to a certain extent, with a larger population required to further differentiate between FFP subtypes.
